# Crystal structure of {2,6-bis­[(di­methyl­amino)­meth­yl]phenyl-κ^3^ *N*,*C* ^1^,*N*′}(bromido/chlorido)­mercury(II)

**DOI:** 10.1107/S2056989017014682

**Published:** 2017-10-20

**Authors:** Anand Gupta, Harkesh B. Singh, Ray J. Butcher

**Affiliations:** a Department of Chemistry, Indian Institute of Technology Bombay, Powai, Mumbai 400 076, India; b Department of Chemistry, Howard University, 525 College Street NW, Washington, DC 20059, USA

**Keywords:** crystal structure, NCN pincer ligands, mercury halide derivatives

## Abstract

In the mol­ecular structure of the title compound, {2,6-bis­[(di­methyl­amino)­meth­yl]phenyl-κ^3^ *N*,*C* ^1^,*N*′}[bromido/chlorido­(0.30/0.70)]mercury(II)–{2,6-bis­[(di­methyl­amino)­meth­yl]phenyl-κ^3^ *N*,*C* ^1^,*N*′}[bromido/chlorido­(0.24/0.76)]mer­cury(II) (1/1), [HgBr_0.30_Cl_0.70_(C_12_H_19_N_2_)]·[HgBr_0.24_Cl_0.76_(C_12_H_19_N_2_)], there are two mol­ecules in the asymmetric unit of formula *L*Hg*X* {*L* = 2,6-bis­[(di­methyl­amino)­meth­yl]phenyl and *X* = Cl/Br}. In each mol­ecule, the halide site is mixed Cl/Br, with occupancies of 0.699 (7):0.301 (7) and 0.763 (7):0.237 (7), respectively. The two mol­ecules are linked into dimers by a combination of Hg⋯Hg [Hg⋯Hg = 3.6153 (3) Å] and C—H⋯Cl and C—H⋯π inter­actions.

## Abstract 2

The structure of C_12_H_19_Br_0.30_Cl_0.70_HgN_2_·C_12_H_19_Br_0.24_Cl_0.76_HgN_2_ contains two mol­ecules in the asymmetric unit with each containing a mixed Cl/Br halide site. The two mol­ecules are linked into dimers by a combination of Hg⋯Hg [Hg⋯Hg = 3.6153 (3) Å] and C—H⋯Cl and C—H⋯π inter­actions.

## Chemical context   

Organomercury compounds of type *R*_2_Hg and *R*Hg*X* (*R* = alkyl or aryl; *X* = halide) have received considerable attention in the last three decades, mainly related to the search for versatile reagents in controlled transmetallation reactions (Wardell, 1985 ▸). Organomercury(II) derivatives have been used successfully to obtain the desired organometallic compounds of transition metals, as well as main group metals otherwise inaccessible by classical Grignard and/or li­thia­tion reactions (Bonnardel *et al.*, 1996 ▸; Gul & Nelson, 1999*a* ▸,*b* ▸; Berger *et al.*, 2001 ▸, 2003 ▸; Zhang *et al.*, 2005 ▸; Djukic *et al.*, 2006 ▸). Although the toxicity of mercury compounds should always be taken into account, there are important advantages, *e.g.* the possibility of preparing functionalized organomercury derivatives and the high selectivity of the transmetallation reaction (Ding *et al.*, 1993 ▸; Pfeffer *et al.*, 1996 ▸; Wu *et al.*, 1998 ▸; Dreher & Leighton, 2001 ▸; Crimmins & Brown, 2004 ▸). Some cyclo­metallated organomercury(II) chlorides containing N-donor functionalized aryl ligands were investigated in the context of their use as transmetallation reagents (Ali *et al.*, 1989 ▸; Constable *et al.*, 1989 ▸, 1991 ▸, Srivastava *et al.*, 2010 ▸). Thus, organomercury(II) compounds serve as the precursor for the synthesis of various organometallic derivatives of transition metals, as well as main group metals, thus there is much inter­est in the structural characterization of these derivatives.

## Structural commentary   

**Figure 1:**
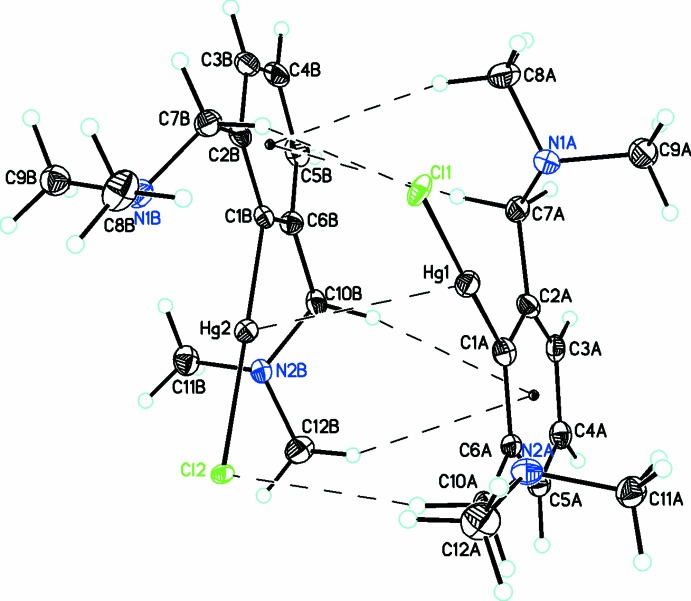
The dimeric unit formed by a combination of Hg⋯Hg, C—H⋯Cl and C—H⋯π inter­actions (all shown with dashed bonds). Only the major chloride moiety is shown. Atomic displacement parameters are at the 30% probability level.

The mol­ecular structure of **2** is shown in Figs. 1 ▸ and 2 ▸. The compound crystallized with two mol­ecules in the asymmetric unit. In each mol­ecule, the halide site is mixed Cl/Br with occupancies of 0.699 (7):0.301 (7) in mol­ecule *A* and 0.763 (7):0.237 (7) in mol­ecule *B*. In these moieties, there are two coordination spheres around each Hg atom (Table 1 ▸). If we consider the first coordination sphere, the spatial arrangement of each Hg atom is distorted square planar with a coordination sphere made up of C—Hg—Cl/Br. Inter­estingly, both amine side arms are displaced from this plane in the same direction and thus both are on the same side of the phenyl ring. This displacement of the bulky groups with respect to the phenyl ring attached to Hg has been observed previously (Lau & Kochi, 1986 ▸).
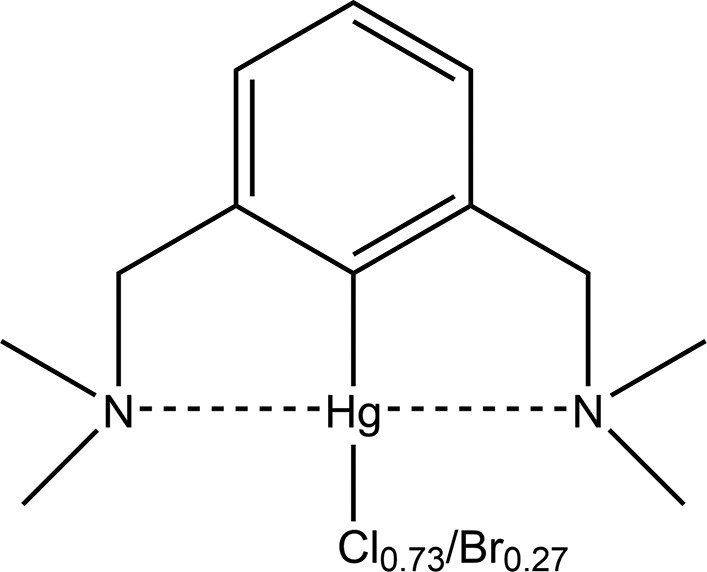


A significant feature of this compound is the presence of a weak inter­action between both chemically similar *d* ^10^–*d* ^10^ metals. The distance of 3.6153 (3) Å is significantly smaller than the sum of the van der Waals radii for Hg⋯Hg (Σ*r*_vdW_ = 3.96 Å; Bondi, 1964 ▸). These inter­molecular inter­actions are longer than the Hg⋯Hg distances reported for metallic mercury (3 Å) (Donohue, 1974 ▸). They are close to the intra­molecular Hg⋯Hg distances observed previously (Hg1⋯Hg2 = 3.572 Å; King *et al.*, 2002 ▸) and also exceed the mercurophilic distances calculated for the (HgMe_2_)_2_ dimer at 3.41 Å (Pyykkö & Straka, 2000 ▸). This is in contrast to a related structure, *L*_2_Hg_2_Br_2_ {*L* = 4-*tert*-butyl-2-[(di­methyl­amino)­meth­yl]-6-[(di­methyl­amino)­meth­yl]benzene}, which is a dimer linked by Hg_2_Br_2_ units, with one di­methyl­amino arm of each ligand coordinated to an Hg atom, and where there are no Hg⋯Hg inter­actions present (Das *et al.*, 2015 ▸). The N⋯Hg distances [Hg1⋯N1*A*/Hg1⋯N2*A* = 2.764 (7)/2.867 (6) Å] are significantly shorter than the sum of the van der Waals radii for Hg and N [Σ*r*_vdW_ (Hg, N) = 3.53 Å]. However, these values are slightly longer than related organo­mercury(II) compounds with one pendant arm of 2.65 (1), 2.725 (4) and 2.647 (2) Å (Attar *et al.*, 1995 ▸; Bumbu *et al.*, 2004 ▸), but are similar to those found in compounds reported previously (Atwood *et al.*, 1983 ▸; Oilunkaniemi *et al.*, 2001 ▸; Zhou *et al.*, 1994 ▸) at 2.787 (6)/2.858 (6) and 2.89 Å.

## Supra­molecular features   

A significant feature of this compound is the presence of a weak inter­action between both chemically similar *d* ^10^–*d* ^10^ metals. The distance is 3.6153 (3) Å, which is significantly smaller than the sum of the van der Waals radii for Hg⋯Hg (Σ*r*_vdW_ =3.96 Å; Bondi, 1964 ▸). This links the mol­ecules into dimers which are further stabilized by both C—H⋯Cl/Br (Table 2 ▸) and C—H⋯π inter­actions, as shown in Figs. 1 ▸ and 2 ▸. In the packing, there are no significant inter­actions apart from those discussed above.

## Database survey   

A survey of the Cambridge Structural Database (Version 5.38; Groom *et al.*, 2016 ▸) for Hg*X* complexes of NCN pincer ligands with each N as a tertiary amine gave four hits: HIMQEA (Spek *et al.*, 2007 ▸), LIGFIS (Liu *et al.*, 2013 ▸), OWUHAQ (Beleaga *et al.*, 2011 ▸) and TUTLOL (Das *et al.*, 2015 ▸).

## Synthesis and crystallization   

The precursor *N*,*C*,*N*-pincer ligand [2,6-(CH_2_NMe_2_)_2_C_6_H_3_Br], **1**, was prepared according to the procedure given by van Koten and co-workers (van de Kuil *et al.*, 1994 ▸) with slight modifications. An excess of HNMe_2_ (in H_2_O) was employed instead of 2.2 equivalents to quench with 2-bromo-1,3-bis­(bromo­meth­yl)benzene. This afforded a yellow oil which was purified by vacuum distillation to give a colorless oil in 70% yield. *n*-BuLi (1.15 ml, 1.84 mmol) was added dropwise *via* syringe to the solution of **1** (0.50 g, 1.84 mmol) in dry Et_2_O (15 ml) under an inert atmosphere at 273 K. After 30 min, the color of the reaction mixture changed from colorless to pale yellow. To this, a solution of HgCl_2_ (0.50 g, 1.84 mmol) in dry THF (10 ml) was added. The whole mixture was stirred for 5 h at 273 K and then allowed to warm slowly to room temperature. Then reaction mixture was filtered and the filtrate evaporated to dryness and the resulting precipitate extracted with hexane. The workup afforded a white precipitate of **2** (yield 0.36 g, 75%; m.p. 408–410 K). Colorless crystals of **2** suitable for single-crystal diffraction analysis were obtained by slow diffusion of hexane into CHCl_3_ at room temperature.

^1^H NMR: δ 7.15 (*t*, 1H, Ar-H), 7.07 (*d*, 2H, ArH), 3.45 (*s*, 4H, CH_2_), 2.21 (*s*, 12H, NCH_3_). ^13^C NMR: δ 144.90, 128.36, 128.10, 66.01, 44.85. ^199^Hg NMR: δ −930. Analysis calculated for C_12_H_19_ClHgN_2_: C 33.73, H 4.48, N 6.56%; found: C 32.55, H 5.10, N 5.26%. ESI–MS (positive mode): [*M* + H]^+^ *m/z* = 429.1005 (observed), 429.1015 (calculated).

## Refinement   

Crystal data, data collection and structure refinement details are summarized in Table 3 ▸. H atoms were positioned geometrically and allowed to ride on their parent atoms, with C—H = 0.95–0.99 Å and *U*_iso_(H) = *xU*_eq_(C), where *x* = 1.5 for methyl H atoms and 1.2 for all other C-bound H atoms. There are two mol­ecules in the asymmetric unit and in each the halide site is occupied by a mix or Cl and Br, with refined occupancies of 0.699 (7):0.301 (7) and 0.763 (7):0.237 (7), respectively.

## Supplementary Material

Crystal structure: contains datablock(s) I. DOI: 10.1107/S2056989017014682/hg5496sup1.cif

Structure factors: contains datablock(s) I. DOI: 10.1107/S2056989017014682/hg5496Isup2.hkl

CCDC reference: 1510433

Additional supporting information:  crystallographic information; 3D view; checkCIF report

## Figures and Tables

**Figure 2:**
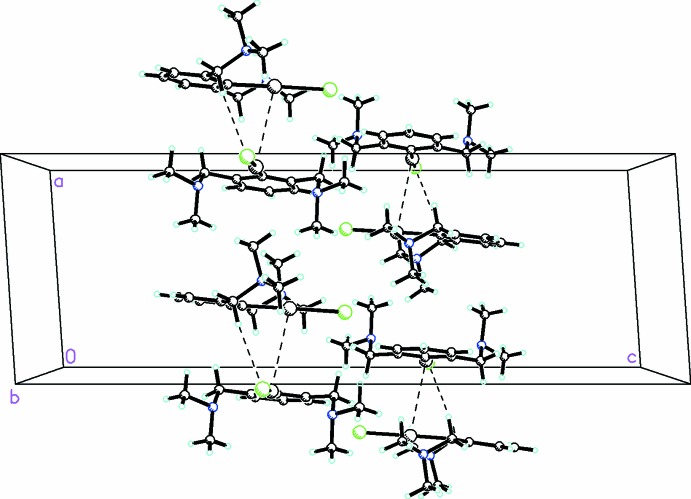
Packing diagram for the title compound, viewed along the *b* axis, showing Hg⋯Hg and C—H⋯Cl inter­actions as dashed lines.

**Table 1:** Selected geometric parameters (Å, °)

Hg1—C1*A*	2.060 (5)	Hg2—C1*B*	2.073 (5)
Hg1—Cl1	2.365 (19)	Hg2—Cl2	2.331 (11)
Hg1—Br1	2.39 (2)	Hg2—Br2	2.417 (19)
Hg1—Hg2	3.6153 (3)		
			
C1*A*—Hg1—Cl1	175.4 (5)	C1*B*—Hg2—Cl2	175.3 (4)
C1*A*—Hg1—Br1	178.6 (6)	C1*B*—Hg2—Br2	178.9 (5)
C1*A*—Hg1—Hg2	82.31 (15)	C1*B*—Hg2—Hg1	86.82 (15)
Cl1—Hg1—Hg2	97.8 (5)	Cl2—Hg2—Hg1	96.7 (3)
Br1—Hg1—Hg2	97.5 (5)	Br2—Hg2—Hg1	92.3 (5)

**Table 2:** Hydrogen-bond geometry (Å, °)

*D*—H⋯*A*	*D*—H	H⋯*A*	*D*⋯*A*	*D*—H⋯*A*
C7*B*—H7*BA*⋯Cl1_a	0.99	2.93	3.90 (2)	167
C7*B*—H7*BA*⋯Br1_b	0.99	3.06	4.01 (2)	163
C10*A*—H10*C*⋯Cl2_a	0.99	2.89	3.871 (16)	171
C10*A*—H10*C*⋯Br2_b	0.99	2.82	3.80 (2)	169
C8*B*—H8*BA*⋯Br1_b^i^	0.98	3.06	4.04 (2)	174
C12*A*—H12*A*⋯Br1_b^ii^	0.98	2.96	3.87 (2)	155

Symmetry codes: (i) 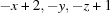; (ii) 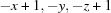.

**Table 3:** Experimental details

Crystal data	
Chemical formula	[HgBr_0.30_Cl_0.70_(C_12_H_19_N_2_)]·[HgBr_0.24_Cl_0.76_(C_12_H_19_N_2_)]
*M*_r_	878.78
Crystal system, space group	Monoclinic, *P*2_1_/*n*
Temperature (K)	123
*a*, *b*, *c* (Å)	9.51872 (15), 10.88545 (17), 27.8353 (5)
β (°)	93.8563 (15)
*V* (Å^3^)	2877.64 (8)
*Z*	4
Radiation type	Cu *K*α
μ (mm^−1^)	21.12
Crystal size (mm)	0.29 × 0.25 × 0.10

Data collection	
Diffractometer	Agilent Xcalibur Ruby Gemini
Absorption correction	Analytical [*CrysAlis PRO* (Agilent, 2012 ▸), based on expressions derived by Clark & Reid (1995 ▸)]
*T*_min_, *T*_max_	0.033, 0.248
No. of measured, independent and observed [*I* > 2σ(*I*)] reflections	11395, 5778, 5120
*R*_int_	0.045
(sin θ/λ)_max_ (Å^−1^)	0.628

Refinement	
*R*[*F* ^2^ > 2σ(*F* ^2^)], *wR*(*F* ^2^), *S*	0.033, 0.075, 1.06
No. of reflections	5778
No. of parameters	317
No. of restraints	12
H-atom treatment	H-atom parameters constrained
Δρ_max_, Δρ_min_ (e Å^−3^)	1.28, −1.66

Computer programs: *CrysAlis PRO* (Agilent, (2012 ▸), *SHELXS97* and *SHELXTL* (Sheldrick, 2008 ▸) and *SHELXL2017* (Sheldrick, 2015 ▸).

## supplementary crystallographic information

### Crystal data

**Table:**

[HgBr_0.30_Cl_0.70_(C_12_H_19_N_2_)]·[HgBr_0.24_Cl_0.76_(C_12_H_19_N_2_)]	*F*(000) = 1654.7
*M**_r_* = 878.78	*D*_x_ = 2.028 Mg m^−^^3^
Monoclinic, *P*2_1_/*n*	Cu *K*α radiation, λ = 1.54184 Å
*a* = 9.51872 (15) Å	Cell parameters from 6238 reflections
*b* = 10.88545 (17) Å	θ = 3.2–75.5°
*c* = 27.8353 (5) Å	µ = 21.12 mm^−^^1^
β = 93.8563 (15)°	*T* = 123 K
*V* = 2877.64 (8) Å^3^	Plate, colorless
*Z* = 4	0.29 × 0.25 × 0.10 mm

### Data collection

**Table:**

Agilent Xcalibur Ruby Gemini diffractometer	5120 reflections with *I* > 2σ(*I*)
Detector resolution: 10.5081 pixels mm^-1^	*R*_int_ = 0.045
ω scans	θ_max_ = 75.7°, θ_min_ = 3.2°
Absorption correction: analytical [CrysAlis PRO (Agilent, 2012), based on expressions derived by Clark & Reid (1995)]	*h* = −11→11
*T*_min_ = 0.033, *T*_max_ = 0.248	*k* = −12→13
11395 measured reflections	*l* = −34→22
5778 independent reflections	

### Refinement

**Table:**

Refinement on *F*^2^	Primary atom site location: structure-invariant direct methods
Least-squares matrix: full	Secondary atom site location: difference Fourier map
*R*[*F*^2^ > 2σ(*F*^2^)] = 0.033	Hydrogen site location: inferred from neighbouring sites
*wR*(*F*^2^) = 0.075	H-atom parameters constrained
*S* = 1.06	*w* = 1/[σ^2^(*F*_o_^2^) + (0.0257*P*)^2^] where *P* = (*F*_o_^2^ + 2*F*_c_^2^)/3
5778 reflections	(Δ/σ)_max_ = 0.002
317 parameters	Δρ_max_ = 1.28 e Å^−^^3^
12 restraints	Δρ_min_ = −1.66 e Å^−^^3^

### Special details

**Table:**

Geometry. All esds (except the esd in the dihedral angle between two l.s. planes) are estimated using the full covariance matrix. The cell esds are taken into account individually in the estimation of esds in distances, angles and torsion angles; correlations between esds in cell parameters are only used when they are defined by crystal symmetry. An approximate (isotropic) treatment of cell esds is used for estimating esds involving l.s. planes.

### Fractional atomic coordinates and isotropic or equivalent isotropic displacement parameters (Å^2^)

**Table:**

	*x*	*y*	*z*	*U*_iso_*/*U*_eq_	Occ. (<1)
Hg1	0.67865 (2)	0.22147 (2)	0.59002 (2)	0.02302 (7)	
Hg2	1.04508 (2)	0.16424 (2)	0.62562 (2)	0.02172 (7)	
Cl1	0.696 (2)	0.217 (2)	0.5057 (7)	0.034 (2)	0.699 (7)
Cl2	1.0246 (15)	−0.0473 (11)	0.6361 (5)	0.0261 (15)	0.763 (7)
Br1	0.693 (2)	0.195 (2)	0.5053 (7)	0.033 (3)	0.301 (7)
Br2	1.008 (2)	−0.0552 (18)	0.6292 (7)	0.028 (3)	0.237 (7)
C7B	1.0827 (6)	0.3309 (6)	0.5309 (2)	0.0293 (12)	
H7BA	0.981756	0.316055	0.521854	0.035*	
H7BB	1.121654	0.379092	0.504766	0.035*	
C3B	1.1228 (6)	0.5310 (6)	0.5743 (2)	0.0308 (12)	
H3BA	1.137928	0.567980	0.544182	0.037*	
C6A	0.6440 (5)	0.1362 (5)	0.69141 (19)	0.0195 (10)	
N2A	0.5517 (5)	0.0024 (5)	0.62484 (17)	0.0254 (9)	
N1A	0.6216 (5)	0.4670 (5)	0.60575 (18)	0.0266 (10)	
C4A	0.6285 (6)	0.2670 (6)	0.7610 (2)	0.0262 (11)	
H4AA	0.616324	0.276087	0.794375	0.031*	
N1B	1.1559 (6)	0.2121 (5)	0.53496 (18)	0.0285 (10)	
N2B	1.1252 (5)	0.2467 (5)	0.71894 (17)	0.0247 (9)	
C4B	1.1289 (7)	0.6030 (6)	0.6159 (3)	0.0333 (13)	
H4BA	1.146235	0.688797	0.614067	0.040*	
C1B	1.0743 (6)	0.3525 (5)	0.6213 (2)	0.0216 (10)	
C7A	0.6960 (6)	0.4705 (5)	0.6536 (2)	0.0275 (11)	
H7AA	0.667054	0.544923	0.670857	0.033*	
H7AB	0.798562	0.476236	0.649976	0.033*	
C9A	0.4755 (7)	0.5018 (6)	0.6083 (3)	0.0364 (14)	
H9AA	0.429645	0.505095	0.575756	0.055*	
H9AB	0.470317	0.582844	0.623472	0.055*	
H9AC	0.427680	0.441186	0.627483	0.055*	
C5B	1.1094 (6)	0.5482 (6)	0.6599 (2)	0.0312 (12)	
H5BA	1.114782	0.596728	0.688286	0.037*	
C3A	0.6472 (5)	0.3708 (5)	0.7327 (2)	0.0252 (11)	
H3AA	0.647103	0.450383	0.746694	0.030*	
C8A	0.6926 (8)	0.5444 (7)	0.5721 (3)	0.0395 (15)	
H8AA	0.657279	0.525039	0.539078	0.059*	
H8AB	0.794228	0.529126	0.575688	0.059*	
H8AC	0.674005	0.630996	0.578908	0.059*	
C10B	1.0566 (6)	0.3661 (6)	0.7115 (2)	0.0258 (11)	
H10A	1.092313	0.423059	0.737239	0.031*	
H10B	0.954042	0.356259	0.714164	0.031*	
C5A	0.6275 (6)	0.1514 (6)	0.7408 (2)	0.0240 (10)	
H5AA	0.615475	0.081382	0.760486	0.029*	
C11B	1.2757 (7)	0.2609 (7)	0.7270 (2)	0.0347 (13)	
H11A	1.321057	0.180288	0.725650	0.052*	
H11B	1.297727	0.297876	0.758692	0.052*	
H11C	1.310550	0.314276	0.702021	0.052*	
C2B	1.0948 (5)	0.4057 (5)	0.5765 (2)	0.0242 (11)	
C1A	0.6658 (6)	0.2400 (5)	0.66324 (19)	0.0223 (10)	
C10A	0.6421 (6)	0.0091 (5)	0.67000 (19)	0.0233 (10)	
H10C	0.739302	−0.015028	0.663499	0.028*	
H10D	0.607285	−0.049896	0.693533	0.028*	
C8B	1.1153 (8)	0.1363 (8)	0.4926 (3)	0.0432 (17)	
H8BA	1.161637	0.056088	0.495918	0.065*	
H8BB	1.144287	0.177335	0.463548	0.065*	
H8BC	1.012946	0.124999	0.490164	0.065*	
C12A	0.5793 (8)	−0.1127 (7)	0.5993 (3)	0.0415 (15)	
H12A	0.527374	−0.112131	0.567652	0.062*	
H12B	0.548508	−0.182616	0.618075	0.062*	
H12C	0.680372	−0.119791	0.595076	0.062*	
C11A	0.4023 (6)	0.0082 (6)	0.6348 (2)	0.0308 (12)	
H11D	0.343974	−0.007355	0.605063	0.046*	
H11E	0.380714	0.089841	0.647181	0.046*	
H11F	0.382401	−0.054127	0.658852	0.046*	
C2A	0.6661 (6)	0.3569 (5)	0.6837 (2)	0.0242 (11)	
C12B	1.0667 (7)	0.1789 (7)	0.7579 (2)	0.0357 (14)	
H12D	1.113371	0.098843	0.761506	0.054*	
H12E	0.965493	0.166659	0.750595	0.054*	
H12F	1.081726	0.225376	0.787987	0.054*	
C6B	1.0819 (6)	0.4219 (5)	0.6630 (2)	0.0231 (10)	
C9B	1.3082 (7)	0.2285 (6)	0.5396 (2)	0.0338 (13)	
H9BA	1.335127	0.269149	0.570282	0.051*	
H9BB	1.337477	0.279205	0.512984	0.051*	
H9BC	1.354303	0.148111	0.538753	0.051*	

### Atomic displacement parameters (Å^2^)

**Table:**

	*U*^11^	*U*^22^	*U*^33^	*U*^12^	*U*^13^	*U*^23^
Hg1	0.02045 (11)	0.02636 (11)	0.02252 (11)	−0.00026 (8)	0.00344 (7)	0.00093 (8)
Hg2	0.02013 (11)	0.02080 (11)	0.02437 (11)	−0.00107 (7)	0.00261 (7)	0.00092 (8)
Cl1	0.037 (2)	0.039 (6)	0.026 (2)	−0.011 (3)	0.0085 (15)	−0.001 (3)
Cl2	0.027 (3)	0.0164 (19)	0.035 (4)	−0.0063 (18)	0.004 (3)	0.000 (2)
Br1	0.029 (3)	0.038 (7)	0.031 (2)	−0.004 (3)	0.0054 (16)	−0.006 (3)
Br2	0.026 (4)	0.024 (3)	0.035 (5)	−0.010 (2)	−0.002 (3)	−0.010 (2)
C7B	0.022 (3)	0.038 (3)	0.028 (3)	−0.002 (2)	−0.002 (2)	0.006 (2)
C3B	0.026 (3)	0.027 (3)	0.040 (3)	0.002 (2)	−0.001 (2)	0.010 (2)
C6A	0.016 (2)	0.019 (2)	0.023 (2)	0.0008 (18)	0.0048 (18)	0.0009 (19)
N2A	0.032 (3)	0.019 (2)	0.025 (2)	−0.0021 (18)	0.0031 (18)	0.0011 (18)
N1A	0.028 (2)	0.025 (2)	0.027 (2)	0.0016 (19)	0.0034 (19)	0.0045 (19)
C4A	0.022 (3)	0.033 (3)	0.024 (2)	0.004 (2)	0.003 (2)	−0.004 (2)
N1B	0.031 (3)	0.031 (3)	0.024 (2)	−0.002 (2)	0.0052 (19)	0.001 (2)
N2B	0.023 (2)	0.026 (2)	0.025 (2)	0.0036 (18)	0.0032 (17)	0.0000 (18)
C4B	0.029 (3)	0.018 (2)	0.053 (4)	0.004 (2)	0.002 (3)	0.006 (3)
C1B	0.019 (2)	0.016 (2)	0.030 (3)	0.0038 (18)	0.005 (2)	0.004 (2)
C7A	0.020 (3)	0.022 (3)	0.040 (3)	−0.004 (2)	0.003 (2)	0.003 (2)
C9A	0.029 (3)	0.030 (3)	0.050 (4)	−0.004 (2)	−0.002 (3)	0.004 (3)
C5B	0.025 (3)	0.029 (3)	0.039 (3)	0.003 (2)	−0.003 (2)	−0.007 (3)
C3A	0.016 (2)	0.025 (3)	0.034 (3)	−0.0020 (19)	0.001 (2)	−0.006 (2)
C8A	0.049 (4)	0.033 (3)	0.038 (3)	0.000 (3)	0.012 (3)	0.013 (3)
C10B	0.020 (2)	0.032 (3)	0.026 (3)	0.007 (2)	0.000 (2)	−0.003 (2)
C5A	0.018 (2)	0.028 (3)	0.026 (3)	−0.002 (2)	−0.0009 (19)	0.001 (2)
C11B	0.029 (3)	0.044 (4)	0.031 (3)	0.006 (3)	0.000 (2)	0.002 (3)
C2B	0.016 (2)	0.027 (3)	0.029 (3)	0.0012 (19)	−0.0017 (19)	0.008 (2)
C1A	0.016 (2)	0.026 (3)	0.024 (2)	−0.0026 (19)	−0.0049 (18)	−0.003 (2)
C10A	0.022 (2)	0.021 (2)	0.027 (3)	0.004 (2)	0.006 (2)	0.000 (2)
C8B	0.043 (4)	0.045 (4)	0.042 (4)	−0.016 (3)	0.010 (3)	−0.015 (3)
C12A	0.053 (4)	0.027 (3)	0.045 (4)	−0.007 (3)	0.007 (3)	−0.014 (3)
C11A	0.025 (3)	0.034 (3)	0.033 (3)	−0.005 (2)	−0.002 (2)	0.000 (2)
C2A	0.020 (2)	0.020 (2)	0.033 (3)	0.0012 (19)	−0.002 (2)	0.000 (2)
C12B	0.036 (3)	0.041 (4)	0.030 (3)	−0.004 (3)	0.004 (2)	0.003 (3)
C6B	0.019 (2)	0.023 (3)	0.027 (3)	0.0039 (19)	0.0000 (19)	0.001 (2)
C9B	0.030 (3)	0.035 (3)	0.037 (3)	0.004 (2)	0.005 (2)	0.008 (3)

### Geometric parameters (Å, º)

**Table:**

Hg1—C1A	2.060 (5)	C7A—H7AB	0.9900
Hg1—Cl1	2.365 (19)	C9A—H9AA	0.9800
Hg1—Br1	2.39 (2)	C9A—H9AB	0.9800
Hg1—Hg2	3.6153 (3)	C9A—H9AC	0.9800
Hg2—C1B	2.073 (5)	C5B—C6B	1.403 (8)
Hg2—Cl2	2.331 (11)	C5B—H5BA	0.9500
Hg2—Br2	2.417 (19)	C3A—C2A	1.396 (8)
C7B—N1B	1.470 (8)	C3A—H3AA	0.9500
C7B—C2B	1.504 (9)	C8A—H8AA	0.9800
C7B—H7BA	0.9900	C8A—H8AB	0.9800
C7B—H7BB	0.9900	C8A—H8AC	0.9800
C3B—C2B	1.392 (8)	C10B—C6B	1.515 (8)
C3B—C4B	1.396 (10)	C10B—H10A	0.9900
C3B—H3BA	0.9500	C10B—H10B	0.9900
C6A—C1A	1.399 (8)	C5A—H5AA	0.9500
C6A—C5A	1.404 (8)	C11B—H11A	0.9800
C6A—C10A	1.506 (7)	C11B—H11B	0.9800
N2A—C11A	1.469 (8)	C11B—H11C	0.9800
N2A—C12A	1.473 (8)	C1A—C2A	1.394 (8)
N2A—C10A	1.477 (7)	C10A—H10C	0.9900
N1A—C9A	1.447 (8)	C10A—H10D	0.9900
N1A—C8A	1.459 (8)	C8B—H8BA	0.9800
N1A—C7A	1.467 (7)	C8B—H8BB	0.9800
C4A—C5A	1.377 (8)	C8B—H8BC	0.9800
C4A—C3A	1.395 (9)	C12A—H12A	0.9800
C4A—H4AA	0.9500	C12A—H12B	0.9800
N1B—C9B	1.458 (8)	C12A—H12C	0.9800
N1B—C8B	1.469 (8)	C11A—H11D	0.9800
N2B—C11B	1.444 (8)	C11A—H11E	0.9800
N2B—C12B	1.454 (8)	C11A—H11F	0.9800
N2B—C10B	1.462 (7)	C12B—H12D	0.9800
C4B—C5B	1.385 (10)	C12B—H12E	0.9800
C4B—H4BA	0.9500	C12B—H12F	0.9800
C1B—C6B	1.383 (8)	C9B—H9BA	0.9800
C1B—C2B	1.400 (8)	C9B—H9BB	0.9800
C7A—C2A	1.531 (8)	C9B—H9BC	0.9800
C7A—H7AA	0.9900		
			
C1A—Hg1—Cl1	175.4 (5)	H8AA—C8A—H8AC	109.5
C1A—Hg1—Br1	178.6 (6)	H8AB—C8A—H8AC	109.5
C1A—Hg1—Hg2	82.31 (15)	N2B—C10B—C6B	112.7 (5)
Cl1—Hg1—Hg2	97.8 (5)	N2B—C10B—H10A	109.1
Br1—Hg1—Hg2	97.5 (5)	C6B—C10B—H10A	109.1
C1B—Hg2—Cl2	175.3 (4)	N2B—C10B—H10B	109.1
C1B—Hg2—Br2	178.9 (5)	C6B—C10B—H10B	109.1
C1B—Hg2—Hg1	86.82 (15)	H10A—C10B—H10B	107.8
Cl2—Hg2—Hg1	96.7 (3)	C4A—C5A—C6A	120.6 (5)
Br2—Hg2—Hg1	92.3 (5)	C4A—C5A—H5AA	119.7
N1B—C7B—C2B	113.7 (5)	C6A—C5A—H5AA	119.7
N1B—C7B—H7BA	108.8	N2B—C11B—H11A	109.5
C2B—C7B—H7BA	108.8	N2B—C11B—H11B	109.5
N1B—C7B—H7BB	108.8	H11A—C11B—H11B	109.5
C2B—C7B—H7BB	108.8	N2B—C11B—H11C	109.5
H7BA—C7B—H7BB	107.7	H11A—C11B—H11C	109.5
C2B—C3B—C4B	120.8 (6)	H11B—C11B—H11C	109.5
C2B—C3B—H3BA	119.6	C3B—C2B—C1B	118.8 (6)
C4B—C3B—H3BA	119.6	C3B—C2B—C7B	119.8 (5)
C1A—C6A—C5A	119.0 (5)	C1B—C2B—C7B	121.3 (5)
C1A—C6A—C10A	121.2 (5)	C2A—C1A—C6A	120.3 (5)
C5A—C6A—C10A	119.8 (5)	C2A—C1A—Hg1	119.7 (4)
C11A—N2A—C12A	109.5 (5)	C6A—C1A—Hg1	119.8 (4)
C11A—N2A—C10A	110.7 (4)	N2A—C10A—C6A	111.9 (4)
C12A—N2A—C10A	109.9 (5)	N2A—C10A—H10C	109.2
C9A—N1A—C8A	111.6 (5)	C6A—C10A—H10C	109.2
C9A—N1A—C7A	110.8 (5)	N2A—C10A—H10D	109.2
C8A—N1A—C7A	110.6 (5)	C6A—C10A—H10D	109.2
C5A—C4A—C3A	120.5 (5)	H10C—C10A—H10D	107.9
C5A—C4A—H4AA	119.7	N1B—C8B—H8BA	109.5
C3A—C4A—H4AA	119.7	N1B—C8B—H8BB	109.5
C9B—N1B—C8B	110.3 (5)	H8BA—C8B—H8BB	109.5
C9B—N1B—C7B	111.3 (5)	N1B—C8B—H8BC	109.5
C8B—N1B—C7B	109.6 (5)	H8BA—C8B—H8BC	109.5
C11B—N2B—C12B	111.4 (5)	H8BB—C8B—H8BC	109.5
C11B—N2B—C10B	110.8 (5)	N2A—C12A—H12A	109.5
C12B—N2B—C10B	111.5 (5)	N2A—C12A—H12B	109.5
C5B—C4B—C3B	119.3 (6)	H12A—C12A—H12B	109.5
C5B—C4B—H4BA	120.3	N2A—C12A—H12C	109.5
C3B—C4B—H4BA	120.3	H12A—C12A—H12C	109.5
C6B—C1B—C2B	121.3 (5)	H12B—C12A—H12C	109.5
C6B—C1B—Hg2	119.4 (4)	N2A—C11A—H11D	109.5
C2B—C1B—Hg2	119.2 (4)	N2A—C11A—H11E	109.5
N1A—C7A—C2A	112.3 (5)	H11D—C11A—H11E	109.5
N1A—C7A—H7AA	109.1	N2A—C11A—H11F	109.5
C2A—C7A—H7AA	109.1	H11D—C11A—H11F	109.5
N1A—C7A—H7AB	109.1	H11E—C11A—H11F	109.5
C2A—C7A—H7AB	109.1	C1A—C2A—C3A	120.1 (5)
H7AA—C7A—H7AB	107.9	C1A—C2A—C7A	120.6 (5)
N1A—C9A—H9AA	109.5	C3A—C2A—C7A	119.2 (5)
N1A—C9A—H9AB	109.5	N2B—C12B—H12D	109.5
H9AA—C9A—H9AB	109.5	N2B—C12B—H12E	109.5
N1A—C9A—H9AC	109.5	H12D—C12B—H12E	109.5
H9AA—C9A—H9AC	109.5	N2B—C12B—H12F	109.5
H9AB—C9A—H9AC	109.5	H12D—C12B—H12F	109.5
C4B—C5B—C6B	120.9 (6)	H12E—C12B—H12F	109.5
C4B—C5B—H5BA	119.6	C1B—C6B—C5B	118.8 (5)
C6B—C5B—H5BA	119.6	C1B—C6B—C10B	121.8 (5)
C4A—C3A—C2A	119.5 (5)	C5B—C6B—C10B	119.3 (5)
C4A—C3A—H3AA	120.2	N1B—C9B—H9BA	109.5
C2A—C3A—H3AA	120.2	N1B—C9B—H9BB	109.5
N1A—C8A—H8AA	109.5	H9BA—C9B—H9BB	109.5
N1A—C8A—H8AB	109.5	N1B—C9B—H9BC	109.5
H8AA—C8A—H8AB	109.5	H9BA—C9B—H9BC	109.5
N1A—C8A—H8AC	109.5	H9BB—C9B—H9BC	109.5
			
C2B—C7B—N1B—C9B	−68.9 (6)	C5A—C6A—C1A—Hg1	−176.4 (4)
C2B—C7B—N1B—C8B	168.9 (5)	C10A—C6A—C1A—Hg1	5.4 (7)
C2B—C3B—C4B—C5B	1.2 (9)	C11A—N2A—C10A—C6A	−72.6 (6)
C9A—N1A—C7A—C2A	79.0 (6)	C12A—N2A—C10A—C6A	166.3 (5)
C8A—N1A—C7A—C2A	−156.7 (5)	C1A—C6A—C10A—N2A	−48.0 (7)
C3B—C4B—C5B—C6B	−0.8 (9)	C5A—C6A—C10A—N2A	133.8 (5)
C5A—C4A—C3A—C2A	−0.5 (8)	C6A—C1A—C2A—C3A	1.2 (8)
C11B—N2B—C10B—C6B	72.6 (6)	Hg1—C1A—C2A—C3A	175.4 (4)
C12B—N2B—C10B—C6B	−162.7 (5)	C6A—C1A—C2A—C7A	177.4 (5)
C3A—C4A—C5A—C6A	−0.6 (8)	Hg1—C1A—C2A—C7A	−8.4 (7)
C1A—C6A—C5A—C4A	1.9 (8)	C4A—C3A—C2A—C1A	0.2 (8)
C10A—C6A—C5A—C4A	−179.8 (5)	C4A—C3A—C2A—C7A	−176.1 (5)
C4B—C3B—C2B—C1B	−0.7 (9)	N1A—C7A—C2A—C1A	43.7 (7)
C4B—C3B—C2B—C7B	177.4 (5)	N1A—C7A—C2A—C3A	−140.1 (5)
C6B—C1B—C2B—C3B	−0.2 (8)	C2B—C1B—C6B—C5B	0.6 (8)
Hg2—C1B—C2B—C3B	−176.2 (4)	Hg2—C1B—C6B—C5B	176.6 (4)
C6B—C1B—C2B—C7B	−178.3 (5)	C2B—C1B—C6B—C10B	178.6 (5)
Hg2—C1B—C2B—C7B	5.8 (7)	Hg2—C1B—C6B—C10B	−5.5 (7)
N1B—C7B—C2B—C3B	136.3 (6)	C4B—C5B—C6B—C1B	−0.1 (9)
N1B—C7B—C2B—C1B	−45.7 (7)	C4B—C5B—C6B—C10B	−178.1 (5)
C5A—C6A—C1A—C2A	−2.2 (8)	N2B—C10B—C6B—C1B	43.1 (7)
C10A—C6A—C1A—C2A	179.5 (5)	N2B—C10B—C6B—C5B	−139.0 (5)

### Hydrogen-bond geometry (Å, º)

**Table:**

*D*—H···*A*	*D*—H	H···*A*	*D*···*A*	*D*—H···*A*
C7*B*—H7*BA*···Cl1_a	0.99	2.93	3.90 (2)	167
C7*B*—H7*BA*···Br1_b	0.99	3.06	4.01 (2)	163
C10*A*—H10*C*···Cl2_a	0.99	2.89	3.871 (16)	171
C10*A*—H10*C*···Br2_b	0.99	2.82	3.80 (2)	169
C8*B*—H8*BA*···Br1_b^i^	0.98	3.06	4.04 (2)	174
C12*A*—H12*A*···Br1_b^ii^	0.98	2.96	3.87 (2)	155

Symmetry codes: (i) −*x*+2, −*y*, −*z*+1; (ii) −*x*+1, −*y*, −*z*+1.
